# Professional Quality of Life of Physicians in Nepal: Addressing the Present-day Need

**DOI:** 10.31729/jnma.4860

**Published:** 2020-03-31

**Authors:** Anju Vaidya, Satish Deo, Shristi Karki, Sudan Prasad Neupane

**Affiliations:** 1 Nepal Health Research Council, Ram Shah Path, Kathmandu, Nepal; 2 Maharajgunj Medical Campus Institute of Medicine, Maharajgunj, Kathmandu, Nepal; 3 Norwegian National Advisory Unit on Concurrent Substance Abuse and Mental Health Disorders, Innlandet Hospital Trust, Brumunddal - 2381, Norway

**Keywords:** *doctors*, *healthcare system*, *job satisfaction*, *Nepal*, *quality of life*

## Abstract

Medical practice is a noble profession that bears with it critical responsibilities on the practitioner and expectations from the public. Few studies originating from low-income countries like Nepal indicate a greater extent of dissatisfaction in relation to workload, financial and social circumstances among doctors leading to lower Quality of life. In addition, there has been a rise in doctor's migration to developed nations in aspire of better living standards and job satisfaction which has aggravated the already resource-constrained healthcare systems in those countries. This challenges both access and equity in healthcare. There are indications, based on first-hand experiences and the socio-political situation, that Quality of Life may be alarmingly poor among doctors working in Nepal. The first step towards a comprehensive effort to addressing this issue would be to carry out researches on doctors to gauge the scale and dimensions of the issue.

## INTRODUCTION

The practice of medicine is a noble profession that brings with it honorand appreciation in society. Despite various work-related challenges such as lengthy career ladder, imbalanced work-family life, competitive professional environment and long work schedule, physicians gain their greatest satisfaction from lifesaving procedures and their ability to help ailing patients. Physicians' job satisfaction plays a vital role in the healthcare system and has become a growing issue of interest.^1^

## PROFESSIONAL QUALITY OF LIFE OF PHYSICIANS

Occupation is one of the important aspects of life. Therefore, there is no doubt that a person's point of view towards his/her work and the experiences he/she has been through is framed by the nature of any changes in his work which will definitely affect the individual's wellbeing, also known as Professional Quality of life (QoL).^2^ In this regard, several studies done in both lowmiddle and high-income countries depict interesting findings regarding QoL of health professionals. Studies done in developed countries, such as in Norway,^3^ indicate that burnout and low level of life satisfaction are highly prevalent among the physicians compared to the general population. Similarly, studies conducted in Asian countries reflect variations in their findings. Physicians working in Lahore^4^ was found to be less satisfied with their job. On the contrary, the overall career satisfaction among Sri Lankan physicians was higher.^5^ Moreover, there is a paucity of data to appreciate the QoL of Nepalese physicians.

## JOB SATISFACTION AMONG PHYSICIANS

Appreciation from people and the community as a whole and working for humanity might be some of the main reasonsforthe satisfaction and commitment of Nepalese physicians towards their job. In addition, financial satisfaction might be high among Nepalese physicians and higher among senior physicians as the work schedule of most of them might not be limited to a single institution and they might be working for longer hours for more money ultimately leading to financial satisfaction. Similar lines of evidence indicated higher level of job satisfaction among physiciansespecially males, above 60 years of age, those working in same institution for more than 5 years and those working in preclinical and para-clinical departments and managerial posts done in middle-income countries such as India.^6^ Similar findings were depicted by the studies done in high-income countries where Norwegian and Icelandic physicians experienced relatively higher level of satisfaction and wellbeing at work.^7^ In contrast, Australian physicians were noted to have belowaverage levels of job satisfaction in association with higher level of exhaustion and difficulty to perform their work efficiently. On the contrary, studies from ten European countries showed influences of doctor's job satisfaction on the healthcare system.^1,2^ Additionally, motivation is also an equally important factor for performance enhancement. Poor QoL and job dissatisfaction of physicians are bound to compromise the work performance. Likewise, it may also encourage physicians to migrate to richer countries for higher income and better living standards.

## SHORTAGE OF PHYSICIANS AND THEIR RISING MIGRATION

The trend of migrating physicians and medical graduates from Nepal is on the rise. This makes the prevailing shortage of physicians, particularly in the remote districts, and health inequalities more pronounced. Similarly, it augments the workload of the physicians working back in the nation. There are various pull and push factors involved in such migration.^8^ Unsurprisingly, factors compromising physicians' QoL and job satisfaction are among key issues influencing this migration. Yet again, it adversely affects the national healthcare system.

## HEALTHCARE SYSTEM AND PATIENT SAFETY

The performance of physiciansis inextricably associated with quality of care and patients' satisfaction.^1^ In addition to efforts being made to provide quality health care to the Nepalese population, the impetus to improve the quality of life of Nepalese physicians also requires priority because negative effects on medical professionals' QoL can lead to a compromise in quality of care provided to the patients.

## CONCLUSION AND RECOMMENDATIONS

QoL and job satisfaction are subjective evaluation which may vary markedly over different settings. Most of the inferences regarding these are deduced from data generated in developed nations. Given the circumstances, the QoL of physicians working in the Nepalese setting is likely to below. The first step towards a comprehensive effort to addressing this issue would be to carry out a national survey of physicians to gauge the scale and dimensions of the issue. It is time to acknowledge physicians' high QoL as a pre-requisite for a quality healthcare system as well as patient safety and satisfaction in the population.

## Conflict of Interest

**None.**
